# Photoswitchable phospholipid FRET acceptor: Detergent free intermembrane transfer assay of fluorescent lipid analogs

**DOI:** 10.1038/s41598-017-02980-x

**Published:** 2017-06-06

**Authors:** Mariko Sumi, Asami Makino, Takehiko Inaba, Yasushi Sako, Fumihiro Fujimori, Peter Greimel, Toshihide Kobayashi

**Affiliations:** 1 Lipid Biology Laboratory, RIKEN, 2-1, Hirosawa, Wako-shi, Saitama 351-0198 Japan; 2 Cellular Informatics Laboratory, RIKEN, 2-1, Hirosawa, Wako-shi, Saitama 351-0198 Japan; 3 Graduate School of Humanities and Life Sciences, Tokyo Kasei University, 1-18-1, Kaga, Itabashi, Tokyo 173-8602 Japan; 4 UMR 7213 CNRS, University of Strasbourg, 67401 Illkirch, France

## Abstract

We have developed and characterized a novel photoswitchable phospholipid analog termed *N*-nitroBIPS-DPPG. The fluorescence can be switched on and off repeatedly with minimal photobleaching by UV or visible light exposure, respectively. The rather large photochromic head group is inserted deeply into the interfacial membrane region conferring a conical overall lipid shape, preference for a positive curvature and only minimal intermembrane transfer. Utilizing the switchable NBD fluorescence quenching ability of *N*-nitroBIPS-DPPG, a detergent free intermembrane transfer assay system for NBD modified lipids was demonstrated and validated. As NBD quenching can be turned off, total NBD associated sample fluorescence can be determined without the need of detergents. This not only reduces detergent associated systematic errors, but also simplifies assay handling and allows assay extension to detergent insoluble lipid species.

## Introduction

Intermembrane lipid transfer is crucial in membrane biogenesis^1, 2^ as well as lipid-mediated signal transduction^3^. Fluorescent lipid analogs have been employed to study intermembrane lipid transport both *in vivo* ^1, 2, 4–6^ and *in vitro* ^2, 7–10^. *In vitro*, Förster resonance energy transfer (FRET) between two fluorescent lipid analogs is often employed to measure lipid transport^7^. In this method, FRET donor and acceptor lipids are incorporated into the same liposome, resulting in the quenching of the donor lipid fluorescence. Mixing with non-fluorescent, quencher free liposomes allow transfer of the donor lipid, which is proportional to an increase in donor lipid fluorescence. At the end of the reaction, detergent is added to disrupt the liposomes and obtain the concentration of the donor lipids in the starting liposomes. However, liposome suspensions tend to be turbid and it is well established that detergent addition affects turbidity. Furthermore, some lipids or lipid mixtures are detergent insoluble. Taken together, these technical difficulties render determination of the final concentration of the energy donor lipids difficult.

Spiropyrans can reversibly switch between the colorless, nonpolar, closed “spiro” form (SP) and the colored, zwitterionic, open “merocyanine” form (MC) upon illumination with visible and UV light, respectively^11, 12^. Photoswitched FRET has been reported between fluorescently labeled DNA and spiropyran^13^ and in spiropyran covalently conjugated to a fluorophore^12^. In the present study, we chemically synthesized a novel phospholipid analog by conjugating an 1,2-dipalmitoyl-*sn*-glycero-3-phosphate with a 2-(3′,3′-dimethyl-6-nitrospiro[1-benzopyran-2,2′-1*H*-indolin]-1′-yl)ethanol head group, herein referred to as *N*-nitroBIPS-DPPG. *N*-nitroBIPS-DPPG exhibits a UV-dependent fluorescence and acts as a photoswitchable FRET acceptor for 7-nitro-1,3-benz-2-oxadiazol-4-yl (NBD)-labeled lipids. Using *N*-nitroBIPS-DPPG, we quantitatively measured spontaneous membrane transfer of three NBD-labeled lipid analogs.

## Results and Discussion

**Figure 1:**
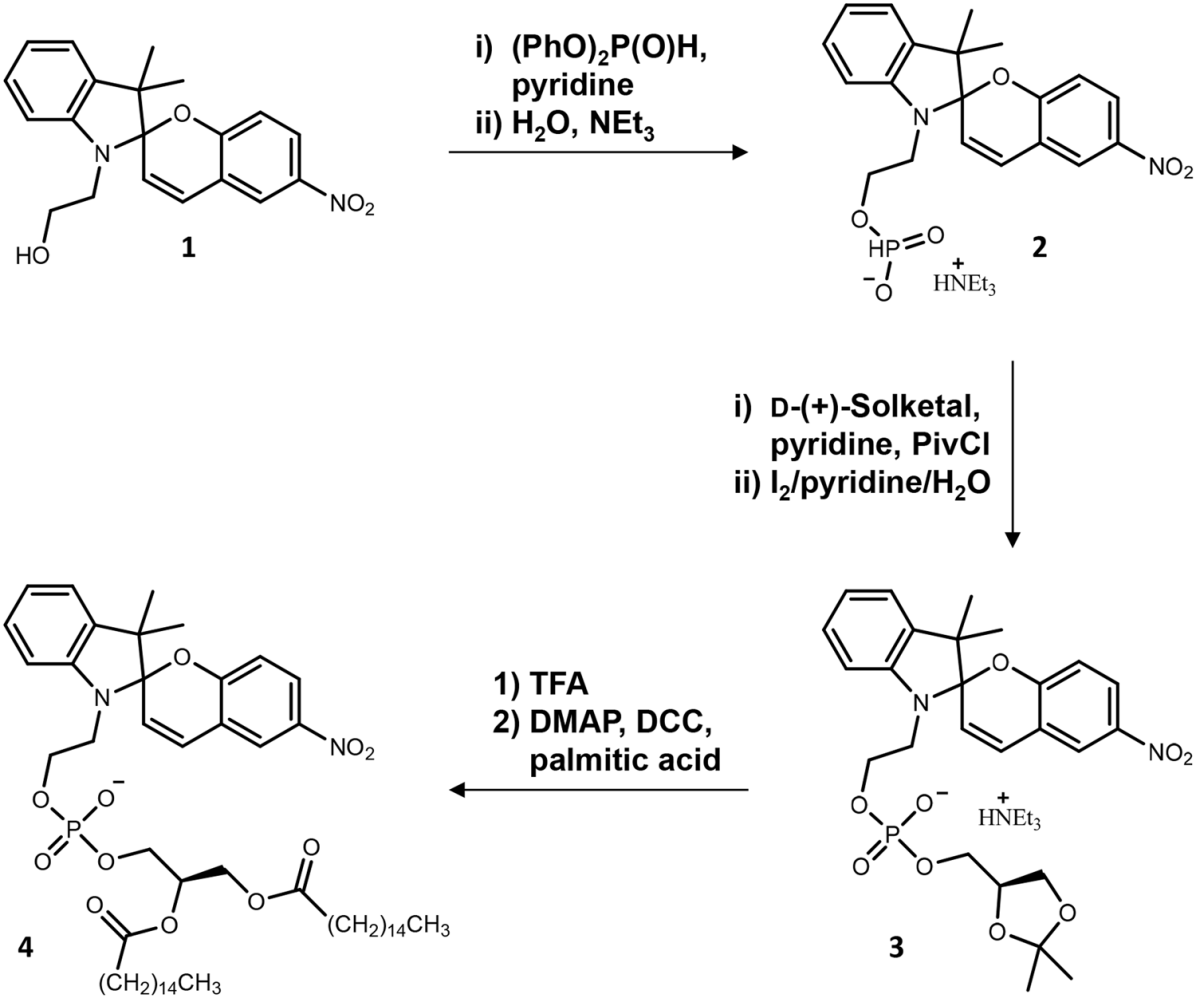
Synthesis of 1,2-dipalmitoyl-sn-glycero-3-phosphoryl-2-(3′,3′-dimethyl-6-nitrospiro[1-benzopyran-2,2′-1*H*-indolin]-1′-yl)ethane (*N*-nitroBIPS-DPPG) (4). 1, 1-(2-hydroxyethyl)-3,3-dimethylindolino-6′-nitrobenzopyrylospiran; 2, triethylammonium 2-(3′,3′-dimethyl-6-nitrospiro [1-benzopyran-2,2′-1*H*-indolin]-1′-yl)ethyl H-phosphonate; 3, triethylammonium ((R)-2,2-dimethyl-1,3-dioxolan-4-yl)methyl (2-(3′,3′-dimethyl-6-nitrospiro[1-benzopyran-2,2′-1*H*-indolin]-1′-yl)ethyl) phosphate.

The synthetic plan towards *N*-nitroBIPS-DPPG is outlined in Fig. 1. Introduction of the fatty acid (FA) residues was chosen as the final step to allow easy alteration of the FA pattern for future work. Additionally, frequently employed 1,2-diacyl-*sn*-glycerol derivatives have been avoided in order to prevent complications due to possible FA migration and its concomitant loss of optical purity during condensation reactions. Shortly, the primary hydroxyl function of commercial *N*-nitroBIPS-ethanol (**1**) was readily converted into the stable H-phosphonate **2**, utilizing diphenyl phosphate^14^. Condensation with d-(+)-solketal was initiated with pivaloyl chloride, followed by immediate oxidation of the unstable P(III) diester to the stable P(V) diester **3**, analogous to previous reports^15, 16^. The isopropylidene protecting group was removed under acidic conditions, monitored by ^1^H-NMR, yielding intermediate **4a**. Subsequently, the crude intermediate was directly acylated with palmitoyl chloride in pyridine overnight. Lastly, the triethylamine counter ion was exchanged after final purification, yielding *N*-nitroBIPS-DPPG. NMR spectra of each compound are shown in Supplementary Information.

**Figure 2:**
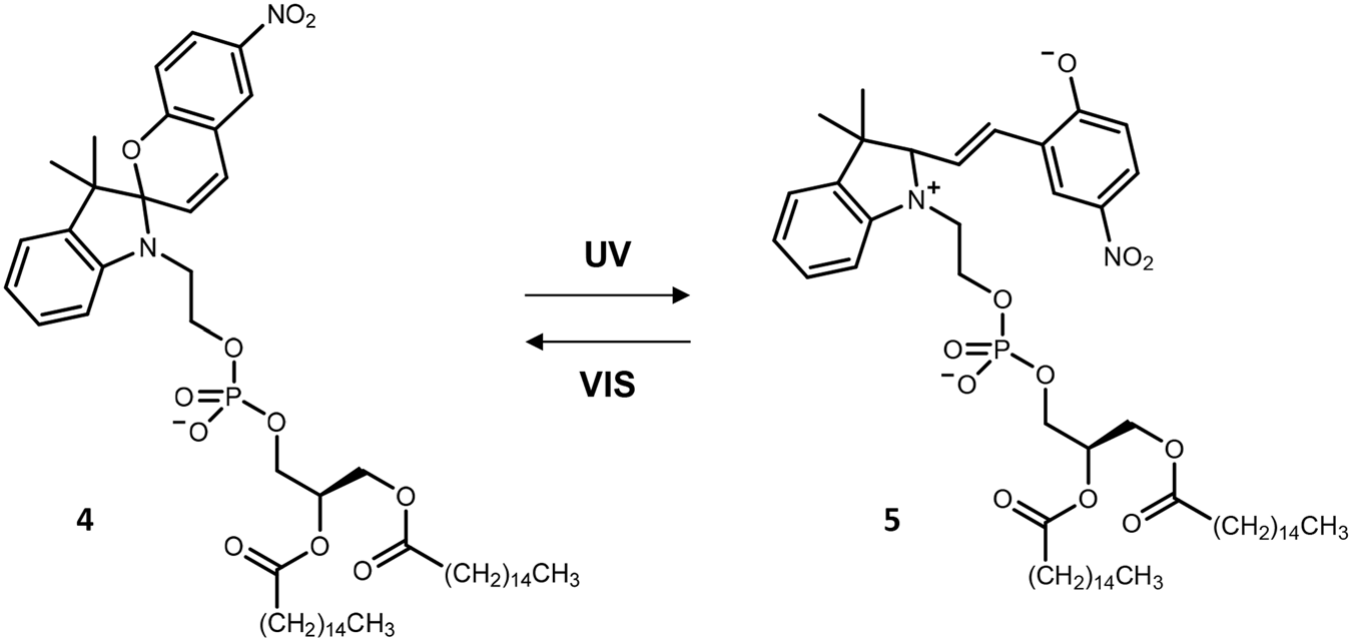
Structural changes of *N*-nitroBIPS-DPPG head group upon exposure to UV or visible light.

In general, *N*-nitroBIPS can exhibit either an SP or MC form (Fig. 2). Importantly, *N*-nitroBIPS-DPPG in the SP form features a single negative charge at the phosphate moiety resembling phosphatidylglycerol (PG). Upon conversion to the MC form, an additional zwitterion pair is formed, allowing interaction of the newly formed positively charged ring nitrogen with the phosphate moiety, resembling phosphatidylcholine (PC). Nevertheless, the total charge of both species, SP and MC, remains negative. Conversion of the SP to MC species of *N*-nitroBIPS is commonly achieved by irradiation with UV light, while the reverse conversion, from MC to SP species, is favored by exposure to visible light.

**Figure 3:**
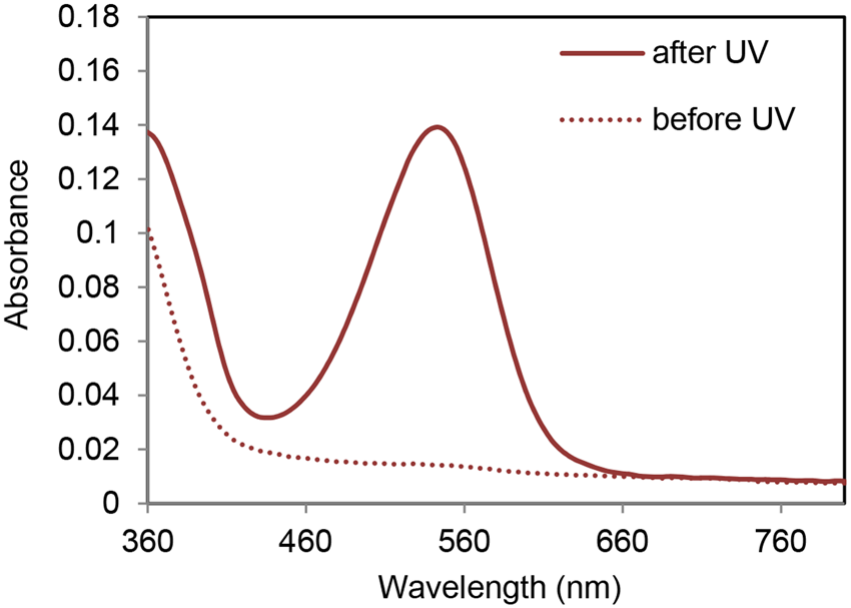
Absorbance spectra of *N*-nitroBIPS-DPPG before and after UV irradiation. The absorption spectra of a DOPC/*N*-nitroBIPS-DPPG (9:1) dispersion (1 mM) in PBS was recorded as described in EXPERIMENTAL SECTION. Representative data of three independent experiments.

The photochemical properties of *N*-nitroBIPS-DPPG were characterized in DOPC: *N*-nitroBIPS-DPPG (9:1) liposome solutions. As expected, the absorption spectra of the SP species of *N*-nitroBIPS-DPPG did not exhibit any absorption beyond ~450 nm (Fig. 3, dotted line). After UV (365 nm) irradiation, *N*-nitroBIPS-DPPG showed MC absorption^17^ with an absorption maximum at 543 nm (Fig. 3, solid line). This confirms that conjugation of *N*-nitroBIPS-ethanol to phospholipids does not impede its photoswitching between the SP and MC species. Additionally, MC is known to exhibit solvatochromism, as reduced solvent polarity and H-bonding capability is associated with a red shift of its absorption maximum from 510 nm to 540 nm and 570 nm in water, ethanol and acetone, respectively^17^. The observed red shift to 543 nm of the *N*-nitroBIPS-DPPG MC moiety suggests that the MC is not exposed to bulk water. The dielectric constant (ε) of ethanol (ε = 25) and acetone (ε = 21) are very similar compared to water (ε = 80), but the two solvents differ strongly in their ability to engage in H-bonding. Computer simulations of solvated DOPC membranes indicate^18^ that an ε similar to ethanol and acetone is present at the depth of the FA ester carbonyl groups. At the same relative membrane depth, the fractional presence of water is ~0.2, indicative of an 80% reduction of H-bonding donor presence in this environment. Interestingly, the H-bond enthalpy in pure ethanol is also ~80% reduced compared to pure water^19^. Together, this suggests that the negatively charged MC moiety is well inserted into the interfacial region of the bilayer, slightly above the carbonyl moieties of the FA esters. It has been previously reported^17, 20^ that free MC preferentially associates with bulk water in the presence of zwitterionic 1-palmitoyl-2-oleoyl-*sn*-glycero-3-phosphocholine (POPC) liposomes, but is able to interact with anionic 1-palmitoyl-2-oleoyl-*sn*-glycero-3-phospho-(1′-*rac*-glycerol) (POPG) liposomes. Under the experimental conditions employed herein, the covalently bound MC moiety of *N*-nitroBIPS-DPPG is rather deeply inserted into the interfacial region of DOPC bilayers. This likely resulted in a relatively large head group size compared to the lipid tail, conferring a more conical overall shape to *N*-nitroBIPS-DPPG.

**Figure 4:**
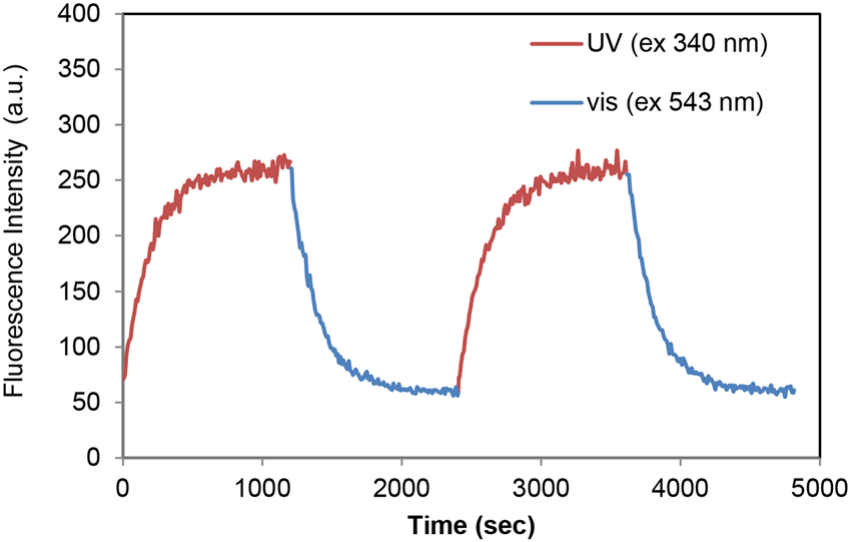
UV dependent photoswitching of *N*-nitroBIPS-DPPG fluorescence. The fluorescence (λex = 543 nm, λem = 600 nm) of a DOPC/*N*-nitroBIPS-DPPG (9:1) dispersion (50 μM) was recorded during alternating (5 sec intervals) UV (340 nm) and green light (543 nm) exposure (red line) and during continuous green light (543 nm) irradiation (blue line). Repeated photoswitching cycles did not reveal significant photobleaching of *N*-nitroBIPS-DPPG fluorescence. Representative data of three independent experiments.

Photoswitching characteristics of *N*-nitroBIPS-DPPG were probed by irradiating DOPC: *N*-nitroBIPS-DPPG liposomes with UV (340 nm) and green light (543), concomitantly, while monitoring fluorescence emission at 600 nm (Fig. 4). The increase in fluorescence during UV exposure is associated with an increased population of the fluorescence active MC species of *N*-nitroBIPS-DPPG. Exposure to visible light (543 nm) only, resulted in a drastic drop of fluorescence emission as *N*-nitroBIPS-DPPG reverted to SP species. Repeated photoswitching cycles did not significantly alter the initially observed switching characteristics. Additionally, photoswitching was also not associated with significant photobleaching, consistent with previous reports on other spiropyran compounds^21, 22^.

**Figure 5:**
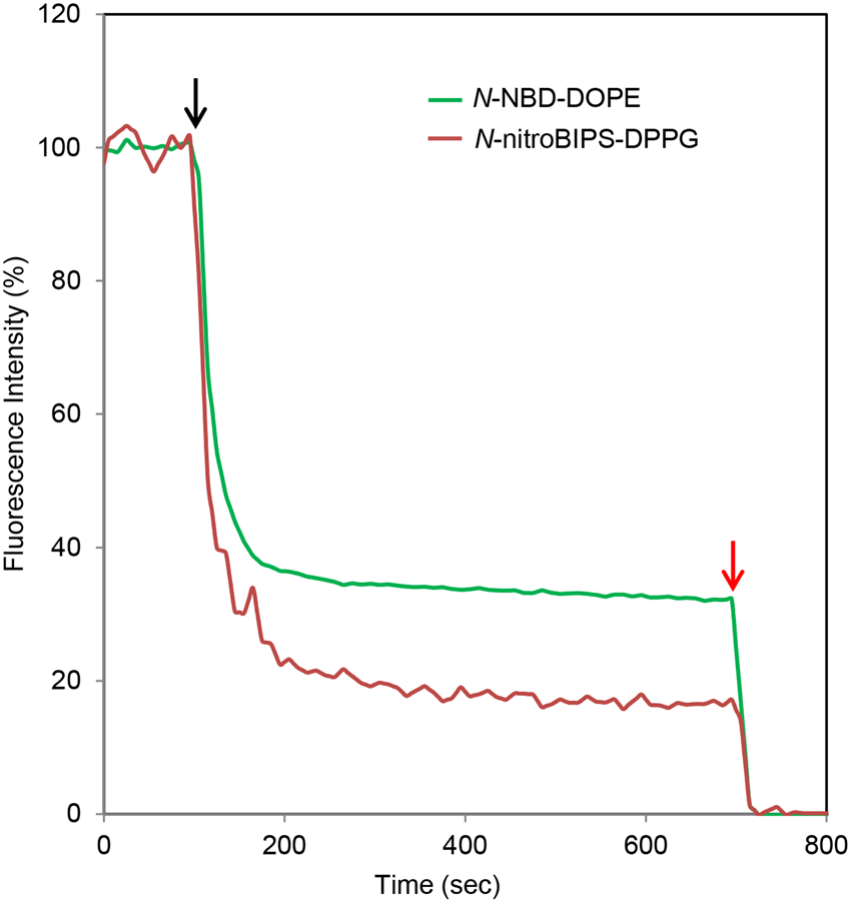
Quenching of outer leaflet *N*-NBD-DOPE (green line) and *N*-nitroBIPS-DPPG (red line). Time course of respective fluorescence emission in DOPC/*N*-nitroBIPS-DPPG/*N*-NBD-DOPE (98:2:2) liposomes as described in EXPERIMENTAL SECTION. Samples were irradiated with UV (340 nm) and blue green (480 nm, NBD) or green (543 nm, nitroBIPS) light alternating every 5 sec. Representative data of three independent experiments. Black arrow, addition of sodium dithionite; red arrow, addition of Triton X-100.

The membrane distribution of *N*-nitroBIPS-DPPG in DOPC liposomes was studied utilizing the membrane-impermeable reducing agent, sodium dithionite. Sodium dithionite has been previously employed to irreversibly reduce nitro groups to amino groups in the outer leaflet of the phospholipid bilayer, effectively inactivating NBD fluorescence^23^. We adopted this method for *N*-nitroBIPS-DPPG quenching, incorporating *N*-NBD-DOPE as control into the liposomes. The time course of NBD and *N*-nitroBIPS fluorescence quenching by sodium dithionite was recorded in independent experiments (Fig. 5). In both cases, the experiment was terminated by addition of Triton X-100 detergent to allow determination of the quenching end point. Nevertheless, addition of detergents reduces solution turbidity by about 10% (see below), complicating quantification of the total range of quenched fluorescence.

During the initial phase, dithionite rapidly quenched NBD as well as *N*-nitroBIPS fluorescence, followed by a slow but steady quenching. The slow fluorescence decrease during extended exposure to the reducing agent is more likely associated with dithionite leakage to the inner leaflet, than to spontaneous flip-flop of the fluorescence emitting probe^24^. NBD fluorescence was reduced to 63% during the initial phase. Taking the decrease in turbidity into account during the final quenching, about 70% of *N*-NBD-DOPE is estimated to be present in the outer leaflet of the liposomes. The diameter of SUVs prepared by ethanol injection has been reported to be ~30 nm^25^. Assuming a bilayer thickness of ~5 nm, the ratio of the surface area between the outer and inner leaflet is 2.25:1. Consequently, ~69% of phospholipids should theoretically be present in the outer SUV leaflet, which is consistent with the corrected experimental results. In contrast to *N*-NBD-DOPE, *N*-nitroBIPS-DPPG fluorescence was reduced by ~80% during the initial quenching phase. Considering the error induced by Triton X-100 reduction of turbidity, nearly 90% of *N*-nitroBIPS-DPPG fluorescence was quenched during the initial phase. Increased leakage of dithionite due to the presence of *N*-nitroBIPS-DPPG can be excluded as *N*-nitroBIPS-DPPG and *N*-NBD-DOPE were incorporated simultaneously in the same liposomes. Similarly, liposome size and thus ratio between inner and outer leaflets were identical between the *N*-nitroBIPS-DPPG and *N*-NBD-DOPE experiments as liposomes from the same preparation were used. Also, the slow second phase quenching rate of *N*-nitroBIPS-DPPG is comparable to the rate exhibited by *N*-NBD-DOPE, suggesting that the spontaneous flip-flop speed of the remaining 10% of *N*-nitroBIPS-DPPG in DOPC membranes might be similarly slow as for *N*-NBD-DOPE. On the one hand, the remaining 10% of fluorescence intensity may be caused by a small population of oligo- or multilamellar liposomes. On the other hand, the conical shape of *N*-nitroBIPS-DPPG and the small radius of the employed SUV preparation could be a driving factor for lipid asymmetry between the inner and outer leaflet. Indeed, computer simulations^26^ suggest that in liposomes with a radius below 60 nm conical shaped lipids favoring positive curvature (large head group with small tail section) tend to be enriched in the outer leaflet, if present at low concentration in a curvature neutral matrix. It was proposed that the degree of enrichment is directly proportional to the ratio of head to tail size in conical lipids. Consequently, quenching of ~90% of *N*-nitroBIPS-DPPG fluorescence would suggest that the head group (including hydration shell) is about 1.5 times larger compared to its lipid tail section. Taken together, the high degree of *N*-nitroBIPS-DPPG quenching due to shape driven lipid asymmetry seems attractive at this point, while rapid flip-flop of *N*-nitroBIPS-DPPG during the initial phase cannot be ruled out.

**Figure 6:**
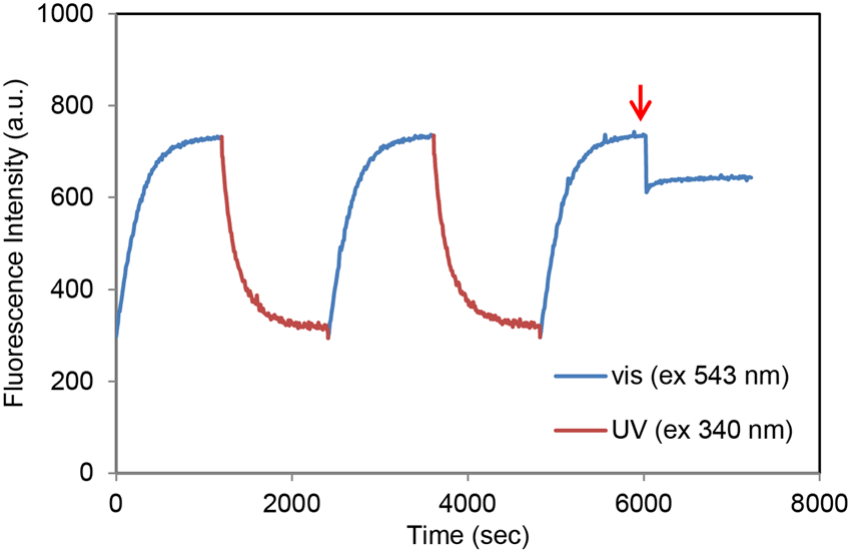
Photoswitchable Förster resonance energy transfer (FRET) between *N*-NBD-DOPE and *N*-nitroBIPS-DPPG. Time course of NBD fluorescence emission of DOPC/*N*-NBD-DOPE/*N*-nitroBIPS-DPPG (89:1:10) liposomes. Representative data of three independent experiments. Blue line, sample was concomitantly irradiated with blue green (480 nm) and green (543 nm) light, deactivating NBD quenching; red line, sample was concomitantly irradiated with blue green (480 nm) and UV (340 nm) light, activating NBD fluorescence quenching; red arrow, indicates addition of Triton X-100.

The close proximity of the NBD fluorescence emission maxima (λem = 535 nm) and the *N*-nitroBIPS-DPPG MC species absorption maxima (λex = 543 nm) suggests that FRET can occur. Indeed, NBD associated fluorescence emission is quenched in DOPC/*N*-nitroBIPS-DPPG/*N*-NBD-DOPE (89:10:1) liposomes concomitantly irradiated with UV and blue green light (480 nm, for excitation of NBD), eventually reaching a plateau (Fig. 6, red line). The remaining low level of NBD fluorescence could be associated with the turbidity of the liposome solution or a distribution differences of the FRET partners between the leaflets. Subsequent switching of *N*-nitroBIPS-DPPG to SP form by visual light excitation abolished NBD quenching (Fig. 6 blue line). This allows repeated on/off cycling of NBD fluorescence quenching without significant photobleaching. Finally, Triton X-100 was added to the solution (Fig. 6, red arrow) while NBD quenching was turned off. Apparently, NBD fluorescence did not increase, confirming complete deactivation of NBD quenching by visual light exposure of *N*-nitroBIPS-DPPG. The reduced NBD fluorescence of about 10-15% is most likely caused by turbidity alteration due to detergent addition, exposing the pitfalls of detergent associated determination of maximal fluorescence.

**Figure 7:**
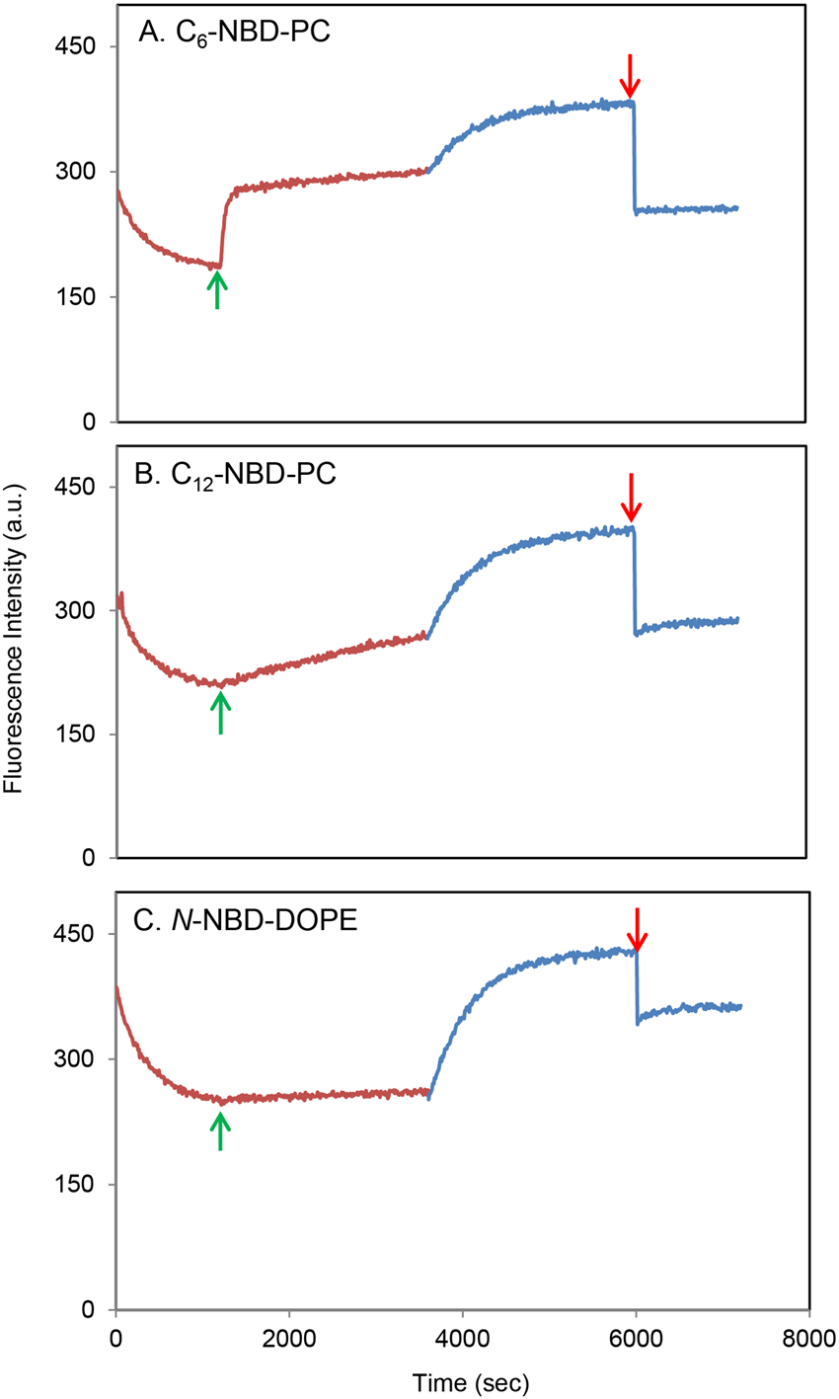
Spontaneous intermembrane transport of NBD lipids, C_6_-NBD-PC (**A**), C_12_-NBD-PC (**B**) and *N*-NBD-DOPE (**C**). Time course of NBD fluorescence emission in DOPC/*N*-nitroBIPS-DPPG/NBD lipid (89:10:1) liposomes Representative data of three independent experiments. Red line, sample was concomitantly irradiated with blue green light (480 nm) and UV light (340 nm), activating NBD fluorescence quenching; blue line, sample was concomitantly irradiated with blue green light (480 nm) and green (543 nm) light, deactivating NBD fluorescence quenching; green arrow, indicates addition of acceptor liposomes; red arrow, indicates addition of Triton X-100.

After characterization of the photochemical properties of *N*-nitroBIPS-DPPG, we established our envisaged detergent free lipid transfer assay. In short, the NBD tagged target lipid is incorporated at an NBD-lipid to *N*-nitroBIPS-DPPG ratio of 1:10 into DOPC (89%) liposomes. Acceptor liposomes composed entirely of DOPC were added to the reaction mixture upon reaching maximum NBD quenching by the UV light induced MC species of *N*-nitroBIPS-DPPG (Fig. 7, green arrow). The increase in NBD fluorescence associated with spontaneous transfer of NBD-lipids to the quencher free liposomes was monitored while maintaining UV light exposure to ensure MC species persistence. To determine total NBD fluorescence, UV light irradiation was switched to visible light irradiation (Fig. 7, blue line), converting *N*-nitroBIPS-DPPG to SP form and abolishing NBD quenching. Only for validation purposes to proof complete abolishment of quenching and to demonstrate the pitfalls of detergent addition Triton X-100 was added at the end of each experiment (Fig. 7, red arrow).

In total, three NBD-labeled phospholipids were selected due to their previously reported specific characteristics^7, 27–30^. *N*-NBD-DOPE was utilized as a negative control due to its known lack of spontaneous transfer between liposomes^27–29^. Short chain FA labeled C_6_-NBD-PC acted as positive control as the combination of short FA and hydrophilic NBD label provides sufficient hydrophilicity to facilitate rapid intermembrane transfer^7, 29, 30^, while the medium chain NBD conjugated FA featuring C_12_-NBD-PC has been reported to exhibit slow intermembrane transfer^7, 29, 30^. Only a marginal fluorescence increase of ~0.1%/min was observed in the presence of *N*-NBD-DOPE during UV irradiation (Fig. 7C). Although this value is nearly 10 times higher than previously reported (0.01%/min)^29^, it is sufficiently slow to allow measurement of the intermembrane transfer rate of other NBD lipids. Additionally, it suggests that *N*-nitroBIPS-DPPG does not exhibits a significant intermembrane transfer speed. The initial transfer rate of C_12_-NBD-PC (Fig. 7B) and C_6_-NBD-PC (Fig. 7A) were determined as 0.77%/min and 80.6%/min, respectively. These values are in good agreement with the 0.6%/min for C_12_-NBD-PC and 94.4%/min for C_6_-NBD-PC reported previously^29^.

Interestingly, in case of C_6_-NBD-PC, after rapid transfer of ~48% of the total NBD fluorescence, a drastic reduction of the fluorescence increase rate to 0.27%/min can be observed. This change could be associated with the establishment of a C_6_-NBD-PC equilibrium between the outer leaflets of the donor and acceptor liposomes, rendering the slow flip-flop of C_6_-NBD-PC from the inner to the outer leaflet of the donor liposome as the rate limiting step. Equilibrium of intermembrane transfer with about 48% of total NBD fluorescence is achieved after 3.8 min. At this point, one can assume that all C_6_-NBD-PC formerly present in the outer leaflet of the donor liposomes is equally spread over the outer leaflet of both donor and acceptor liposomes. Additionally, a maximum of 1% of C_6_-NBD-PCs has undergone spontaneous flip-flop from the inner to the outer leaflet of the donor liposomes. Furthermore, the donor and acceptor liposome preparations are likely to exhibit nearly identical size distributions as both are DOPC-based SUVs. As the double amount of acceptor liposomes compared to donor liposomes were added, the recorded C_6_-NBD-PC signal of 48% corresponds to 2/3 of the C_6_-NBD-PC population present in the outer leaflet of the donor liposomes at the start of the experiment. This suggests that 72% of C_6_-NBD-PC was initially incorporated into the outer leaflet of the donor liposomes. This is in good agreement with the above mentioned presence of *N*-NBD-DOPE and theoretical distribution calculations, indicating that neither *N*-NBD-DOPE nor C_6_-NBD-PC experience significant asymmetric enrichment.

## Conclusion

Taken together, *N*-nitroBIPS-DPPG is a novel UV-light dependent photoswitchable lipid analogue that can readily be incorporated into liposomes. The MC moiety is rather deep inserted into the interfacial region, close to the FA carbonyl oxygens, resulting in a rather large head group size compared to its saturated FA tail. Its suitability as a switchable FRET acceptor and quencher of NBD fluorescence has been demonstrated. Utilizing *N*-nitroBIPS-DPPG switchable quenching, a simplified and improved intermembrane lipid transfer assay has been validated. Importantly, as quenching can be switched off, the need to add detergent, such as Triton X-100 at the end of the experiment to determine maximal fluorescence can be omitted. This renders corrections for turbidity changes caused by the detergent obsolete and significantly reduces systematic errors.

## Materials and Methods

### Materials

1,2-dioleoyl-*sn*-glycero-3-phosphocholine (DOPC), 1,2-dioleoyl-*sn*-glycero-3-phosphoethanolamine-*N*-(7-nitro-1,3-benz-2-oxadiazol-4-yl) ammonium salt (*N*-NBD-DOPE), 1-palmitoyl-2-{6-[(7-nitro-1,3-benz-2-oxadiazol-4-yl) amino] hexanoyl}-*sn*-glycero-3-phosphocholine (C_6_-NBD-PC) were from Avanti Polar Lipids (Alabaster, AL). 1-palmitoyl-2-{12-[(7-nitro-1,3-benz-2-oxadiazol-4-yl) amino] dodecanoyl}-*sn*-glycero-3-phosphocholine (C_12_-NBD-PC) was from Molecular Probes (Eugene, OR). Sodium dithionite was from Sigma (St. Louis, MO).

### Chemical synthesis of *N*-nitroBIPS-DPPG

Unless stated otherwise, reactions were performed under argon, all solvents and chemicals were purchased as reagent grade from commercial suppliers and used without further purification. Dry solvents were purchased from Kanto Chemical Co., Inc. and used as supplied. Analytical thin layer chromatography (TLC) and flash column chromatography were performed using the indicated solvent systems on Merck silica gel 60 F256 plates and Kanto Chemical Co., Ltd. silica gel 60 N (40-100 mesh) respectively. NMR spectra were obtained on a JEOL ECA-500 spectrometer (^1^H at 500, ^13^C at 125 MHz, ^31^P at 200 MHz) in the indicated solvents, with chemical shift referenced to residual non-deuterated solvent.

### Triethylammonium 2-(3′,3′-dimethyl-6-nitrospiro [1-benzopyran-2,2′-1*H*-indolin]-1′-yl)ethyl H-phosphonate (2)

1-(2-Hydroxyethyl)-3,3-dimethylindolino-6′-nitrobenzopyrylospiran (**1**, 100 mg, 0.28 mmol) were dissolved in pyridine, anh. (1 mL) and cooled to 0 °C. After addition of diphenyl phosphate (200 mg, 0.85 mmol) the reaction was stirred for 1 h while slowly reaching RT. Subsequently, triethylamine (73 mg, 0.72 mmol) and water, dist (100 mg, 5.55 mmol) were added and the reaction mixture was stirred for 1 h at RT. The volume was increased with dichloromethane (5 mL) prior to extraction against HCl, dil. The organic layer was dried over Na_2_SO_4_ and concentrated in vacuo. The residue was subjected to flash chromatography on silica gel (gradient of CHCl_3_:MeOH = 1:0 to 0:1 containing 1% NEt_3_), product elution was monitored by TLC (CHCl_3_:MeOH:NEt_3_ = 5:1 + 1%), to give compound **2** as an orange amorphous solid (145 mg, 0.28 mmol, 99% yield). ^1^H NMR (CDCl_3_, 25 °C): δ = 7.99 (dd, 1 H, J = 8.6, J = 2.9, BPy H-7), 7.97 (d, 1 H, J = 2.3, BPy H-5), 7.16 (ddd, 1 H, J = 7.9, J = 7.3, J = 1.6, Ind H-5), 7.06 (dd, 1 H, J = 7.4, J = 1.1, Ind H-6), 6.87 (d, 1 H, J = 10.3, BPy H-4), 6.84 (d, 1 H, J = 7.4, Ind H-4), 6.81 (d, 1 H, J = 616., PH), 6.73 (d, 1 H, J = 8.6, BPy H-8), 6.71 (d, 1 H, J = 7.4, Ind H-7), 5.95 (d, 1 H, J = 10.3, BPy H-3), 3.98 (m, 2 H, Et H-1a/b), 3.49 (m, 1 H, Et H-2a), 3.39 (m, 1 H, Et H-2b), 3.04 (q, 6 H, J = 7.2, NEt_3_ CH_2_), 1.32 (t, 9 H, J = 7.2, NEt_3_ CH_3_), 1.26 (s, 3 H, Me), 1.16 (s, 3 H, Me); ^13^C NMR (CDCl_3_, 25 °C): δ = 159.7 (1 C, BPy C-8a), 147.1 (1 C, Ind C-7a), 141 (1 C, BPy C-6), 135.9 (1 C, Ind C-3a), 128.1 (1 C, Ind C-4), 127.9 (1 C, Ind C-6), 125.9 (1 C, BPy C-3), 122.8 (1 C, BPy C-7), 122.4 (1 C, BPy C-5), 121.7 (1 C, BPy C-4), 119.7 (1 C, BPy C-4a), 118.8 (1 C, Ind C-5), 115.6 (1 C, C-spiro), 107 (1 C, BPy C-8), 106.8 (1 C, Ind C-7), 61.8 (1 C, d, J = 3.6, Et C-1), 52.9 (1 C, Ind C-3), 50.9 (1 C, Et C-2), 45.6 (3 C, NEt_3_ C-1), 26.1 (1 C, Me), 20 (1 C, Me), 8.7 (3 C, NEt_3_ C-2); ^31^P NMR (CDCl_3_, 25 °C): δ = 5.44 (1 P, PHO_3_).

### Triethylammonium ((R)-2,2-dimethyl-1,3-dioxolan-4-yl)methyl (2-(3′,3′-dimethyl-6-nitrospiro[1-benzopyran-2,2′-1*H*-indolin]-1′-yl)ethyl) phosphate (3)

H-Phosphonate **2** (88 mg, 0.17 mmol) and (S)-(+)-2,2-dimethyl-1,3-dioxolane-4-methanol (34 mg, 0.26 mmol) were dissolved in a mixture of tetrahydrofuran, anh. (2 mL) and pyridine (0.69 mL, 8.5 mmol) and cooled to 0 °C. The condensation was initiated by addition of pivaloyl chloride (0.053 mL, 0.425 mmol), immediately heated to RT and stirred vigorously for 10 min. Oxidation was initiated by addition of a iodine/pyridine/water mixture (1.73 mL, 0.196 M iodine in pyridine/water 95:5) and the mixture was stirred for 30 min at RT. The volume was increased with chloroform (40 mL) prior to extraction against aq Na_2_S_2_O_3_ solution (1 M). The organic layer was washed with NEt_3_/H_2_CO_3_-buffer (1 M, pH = 8.4), dried over Na_2_SO_4_ and concentrated in vacuo. The residue was subjected to flash chromatography on silica gel (gradient of CHCl_3_:MeOH = 1:0 to 5:1 containing 1% NEt_3_), product elution was monitored by TLC (CHCl_3_:MeOH:NEt_3_ = 5:1 + 1%), to give compound **3** as an orange amorphous solid (104 mg, 0.16 mmol, 94% yield). ^1^H NMR (CDCl_3_, 25 °C): δ = 7.96 (dd, 1 H, J = 8.9, J = 2.6, BPy H-7), 7.92 (dd, 1 H, J = 2.9, J = 2.3, BPy H-5), 7.09 (dd, 1 H, J = 7.4, J = 7.4, Ind H-5), 7.04 (d, 1 H, J = 7.4, Ind H-6), 6.85-6.80 (n.r., 2 H, BPy H-4, Ind H-4), 6.68 (d, 1 H, J = 9.2, BPy H-8), 6.64 (d, 1 H, J = 6.9, Ind H-7), 5.87 (d, 1 H, J = 10.3, BPy H-3), 4.10 (m, 1 H, Gly H-3a), 3.93 (m, 2 H, Et H-1a/b), 3.81-3.52 (n.r., 4 H, Gly H-3b/2/1a/1b), 3.44 (m, 1 H, Et H-2a), 3.31 (m, 1 H, Et H-2b), 2.97 (q, 6 H, J = 7.4, NEt_3_ CH_2_), 1.29 (s, 3 H, iPr CH_3_), 1.28 (s, 3 H, iPr CH_3_), 1.25 (t, 9 H, J = 7.4, NEt_3_ CH_3_), 1.10 (s, 3 H, Me), 1.09 (s, 3 H, Me); ^13^C NMR (CDCl_3_, 25 °C): δ = 159.5 (1 C, BPy C-8a), 146.9 (1 C, Ind C-7a), 141.1 (1 C, BPy C-6), 135.8 (1 C, Ind C-3a), 128.2 (1 C, Ind C-4), 127.9 (1 C, Ind C-6), 125.9 (1 C, BPy C-3), 122.8 (1 C, BPy C-7), 122.3 (1 C, BPy C-5), 121.8 (1 C, BPy C-4), 119.9 (1 C, BPy C-4a), 118.7 (1 C, Ind C-5), 115.5 (1 C, C-spiro), 109.6 (1 C, BPy C-8), 106.9 (1 C, Ind C-7), 106.8 (1 C, iPr C_quart_), 74.8 (2 C, Et C-1, Gly C-2), 66.3 (2 C, Gly C-1/3), 52.9 (1 C, Ind C-3), 45.5 (3 C, NEt_3_ C-1), 27.4 (1 C, iPr CH_3_), 26.7 (1 C, Me), 25.9 (1 C, Et C-2), 25.2 (1 C, iPr CH_3_), 20 (1 C, Me), 8.8 (3 C, NEt_3_ C-2); ^31^P NMR (CDCl_3_, 25 °C): δ = 1.16 (1 P, PHO_3_).

### 1,2-dipalmitoyl-sn-glycero-3-phosphoryl-2-(3′,3′-dimethyl-6-nitrospiro[1-benzopyran-2,2′-1*H*-indolin]-1′-yl)ethane (4)

Phosphate **3** (85 mg, 0.13 mmol) was suspended in trifluoroacetic acid (1 mL) and stirred at RT for 1 min. Subsequently, the solvent was shortly reduced under a flow of nitrogen prior to suspension in toluene (10 mL). The resulting mixture was concentrated *in vacuo*, followed by two subsequent co-evaporations with toluene (each 10 mL). Complete removal of the isopropylidene group in crude intermediate **4a** (40 mg, 0.07 mmol) was confirmed by ^1^H-NMR. ^1^H NMR (CDCl_3_, 25 °C): δ = 8.01 (d, 1 H, J = 2.9, BPy H-7), 7.98 (dd, 1 H, J = 6.3, J = 2.3, BPy H-5), 7.16 (ddd, 1 H, J = 8., J = 7.4, J = 1.1, Ind H-5), 7.06 (dd, 1 H, J = 7.4, J = 1.1, Ind H-6), 6.89 (d, 1 H, J = 7.2, BPy H-4), 6.86 (ddd, 1 H, J = 7.4, J = 7.4, J = 1.1, Ind H-4), 6.74 (d, 1 H, J = 9.2, BPy H-8), 6.71 (d, 1 H, J = 8., Ind H-7), 5.94 (d, 1 H, J = 10.3, BPy H-3), 4.05-3.86 (m, 4 H, Et H-1a/b, Glc H-3a/b), 3.76 (n.r., 1 H, Gly H-2), 3.64-3.56 (n.r., 2 H, Gly H-1a/b), 3.51 (m, 1 H, Et H-2a), 3.42 (m, 1 H, Et H-2b), 2.96 (q, 6 H, J = 7.3, NEt_3_ CH_2_), 1.26 (s, 3 H, Me), 1.25 (t, 9 H, J = 7.2, NEt_3_ CH_3_), 1.16 (s, 3 H, Me).

Crude intermediate **4a** (40 mg, 0.7 mmol) was dissolved in pyridine (3 mL) and treated with palmitoyl chloride (0.15 mL, 0.48 mmol) at RT and stirred overnight. The resulting slurry was twice co-evaporated with toluene (each 10 mL) and subjected to flash chromatography on silica gel (gradient of CHCl_3_:MeOH = 1:0 to 100:1 containing 1% NEt_3_), product elution was monitored by TLC (CHCl_3_:MeOH:NEt_3_ = 100:1 + 1%), to give compound **4** as an orange amorphous solid (30 mg, 0.16 mmol, 21% yield over two steps). Finally, the majority of the triethylamine counter ion was removed by passing compound 4 over a short silica gel column utilizing CHCl3:MeOH = 1:1 as eluent. Efficiency of counter ion removal was evaluated by ^1^H NMR and any residual presence of triethylamine was well below 5%. ^1^H-NMR. ^1^H NMR (CDCl_3_, 25 °C): δ = 7.95 (dd, 1 H, J = 3.4, J = 2.9, BPy H-7), 7.94 (s, 1 H, BPy H-5), 7.11 (dd, 1 H, J = 7.4, J = 7.4, Ind H-5), 7.02 (d, 1 H, J = 6.9, Ind H-6), 6.88 (d, 1 H, J = 9.7, BPy H-4), 6.80 (dd, 1 H, J = 7.4, J = 7.4, Ind H-4), 6.66 (d, 1 H, J = 9.7, BPy H-8), 6.64 (d, 1 H, J = 8., Ind H-7), 5.89 (dd, 1 H, J = 10.3, J = 1.7, BPy H-3), 5.14 (m, 1 H, Gly H-2), 4.54 (d, 1 H, Gly H-1a), 4.24 (d, 1 H, J = 10.9, Gly H-1b), 3.99 (m, 1 H, Gly H-3a), 3.89 (m, 1 H, Et H-1a), 3.80-3.72 (n.r., 2 H, Gly H-3b, Et H-1b), 3.44 (m, 1 H, Et H-2a), 3.30 (m, 1 H, Et H-2b), 2.20 (m, 4 H, 2xFA H-2a/b), 1.50 (n.r., 4 H, 2xFA H-3a/b), 1.30-1.22 (n.r., 48 H, FA), 1.10 (s, 3 H, Me), 0.87 (t, 6 H, 2xFA CH_3_), 0.87 (s, 3 H, Me); ^31^P NMR (CDCl_3_, 25 °C): δ = 2.07 (1 P, PHO_3_).

### Preparation of liposomes

Small unilamellar vesicles (SUVs) composed of DOPC/*N*-nitroBIPS-DPPG (9:1), DOPC/*N*-nitroBIPS-DPPG/*N*-NBD-DOPE (98:2:2), DOPC/*N*-nitroBIPS-DPPG/NBD lipid (*N*-NBD-DOPE or C_6_-NBD-PC or C_12_-NBD-PC) (89:10:1) were prepared as described previously^25, 31^ at a total lipid concentration of 1 mM. In brief, lipids were aliquoted from chloroform stock solution, dried under N_2_ gas and solvent traces were removed under vacuum for 1 h. The resulting lipid film was dissolved in ethanol, injected into PBS (pH 7.4) (final ethanol concentration 7.5%) under vortex mixing and dialyzed against PBS for 24 h at 4 °C to remove the ethanol. DOPC lipid films were suspended in PBS (5 mM DOPC final concentration), subjected to three freeze-thaw cycles followed by sonication (Branson Sonifier Model 250, Emerson Electric Co., St. Louis, MO) for 10 min.

### Measurement of *N*-nitroBIPS-DPPG absorption spectra

The absorption spectra of 1 mM DOPC/*N*-nitroBIPS-DPPG (9:1) were measured using a UV-visible spectrophotometer (V-650, JASCO, Tokyo) before and after UV irradiation at room temperature. UV irradiation was carried out for 5 min on a UV transilluminator (365 nm, AE-6911CX, ATTO, Tokyo).

### Measurement of *N*-nitroBIPS-DPPG fluorescence

The fluorescence characteristics (λex = 543 nm, λem = 600 nm) of a 50 μM (total lipid) DOPC/*N*-nitroBIPS-DPPG (9:1) liposome solution in PBS was monitored on a spectrofluorometer (FP-6500, JASCO) under constant mixing at room temperature. First, UV (340 nm) and green light (543 nm) were alternately irradiated every 5 sec. During green light irradiation, fluorescence emission at λem = 600 nm was monitored. After maximal fluorescence was attained, the irradiation scheme was switched to continuous irradiation with green light and simultaneous fluorescence detection at λem = 600 nm. Upon return to minimal fluorescence, the irradiation scheme was switched back to the initial scheme and the above detailed cycle was repeated.

### Measurement of *N*-nitroBIPS-DPPG transbilayer membrane asymmetry

DOPC/*N*-nitroBIPS-DPPG/*N*-NBD-DOPE (98:2:2) liposomes were prepared as outlined above and diluted to the final concentration 50 μM with Tris-HCl buffer (50 mM, pH 9.0). Under constant mixing at room temperature, the sample was alternately irradiated every 5 sec with UV (340 nm) and blue green (480 nm) or green (543 nm) light in a spectrofluorometer (FP-6500, JASCO). Upon reaching maximal *N*-nitroBIPS-DPPG fluorescence, 20 μL of 1 M sodium dithionite (final concentration 10 mM) was injected into the liposome suspension^23, 32^. Finally, 20 μL of 10% Triton X-100 (final concentration 0.1%) were added after stable fluorescence emission was detected. The time course of *N*-NBD-DOPE (λex = 480 nm, λem = 535 nm) and *N*-nitroBIPS-DPPG (λex = 543 nm, λem = 600 nm) fluorescence emission was followed in a separate experiments.

### Förster resonance energy transfer (FRET) between *N*-NBD-DOPE and *N*-nitroBIPS-DPPG

50 μM (total lipids) DOPC/*N*-nitroBIPS-DPPG/*N*-NBD-DOPE/(89:10:1) liposomes were alternately irradiated with blue green (480 nm) and green (543 nm) light every 5 sec during constant mixing at room temperature. During the whole experiment the NBD fluorescence signals were monitored at λem = 535 nm during blue green light irradiation. Upon attaining saturation of the fluorescence signal, the irradiation scheme was switched and the sample was alternately irradiated with UV (340 nm) and blue green light every 5 sec. After the fluorescence signal stabilized at minimal levels, the irradiation scheme was switched back to the initial scheme and above outlined irradiation cycle was repeated. As indicated, 20 μL of 10% Triton-X 100 (final concentration 0.1%) solution was added to the liposomes mixture during alternating blue green and green light irradiation.

### Intermembrane transport of NBD lipids

50 μM (total lipids) DOPC/*N*-nitroBIPS-DPPG/NBD lipid (*N*-NBD-DOPE or C_6_-NBD-PC or C_12_-NBD-PC) (89:10:1) liposomes were alternately irradiated with UV (340 nm) and blue green light (480 nm) every 5 sec during constant mixing at room temperature. During the whole experiment fluorescence emission at λem = 535 nm was recorded during blue green light irradiation. After the fluorescence signal stabilized at minimal levels, DOPC liposomes (100 μM total lipids) were added. As indicated, the sample was alternately irradiated with blue green (480 nm) and green (543 nm) light every 5 sec while maintaining constant mixing at room temperature. As marked, 20 μL of a 10% Triton-X 100 (final concentration 0.1%) solution were added to the liposomes.

## Electronic supplementary material

Supplementary Information
