# Supplementary material for: Photoswitchable phospholipid FRET acceptor: Detergent free intermembrane transfer assay of fluorescent lipid analogs
Source: Sci Rep. 2017 Jun 6;7:2900. doi: 10.1038/s41598-017-02980-x (PMC5460167; doi:10.1038/s41598-017-02980-x)
Supplement: Supplementary file 1 — Supplementary Information [file 41598_2017_2980_MOESM1_ESM.pdf]

Supplementary Information

Photoswitchable phospholipid FRET acceptor: Detergent free intermembrane transfer assay of fluorescent lipid analogs

Mariko Sumi, Asami Makino, Takehiko Inaba, Yasushi Sako, Fumihiro Fujimori, Peter Greimel, Toshihide Kobayashi

Table of Contents

NMR spectra of compound 2 2

NMR spectra of compound 3 3

NMR spectra of intermediate 4a 5

NMR spectra of compound 4 5

Triethylammonium 2-(3',3'-dimethyl-6-nitrospiro [1-benzopyran-2,2’-1*H*-indolin]-1'-yl)ethyl H-phosphonate (2)

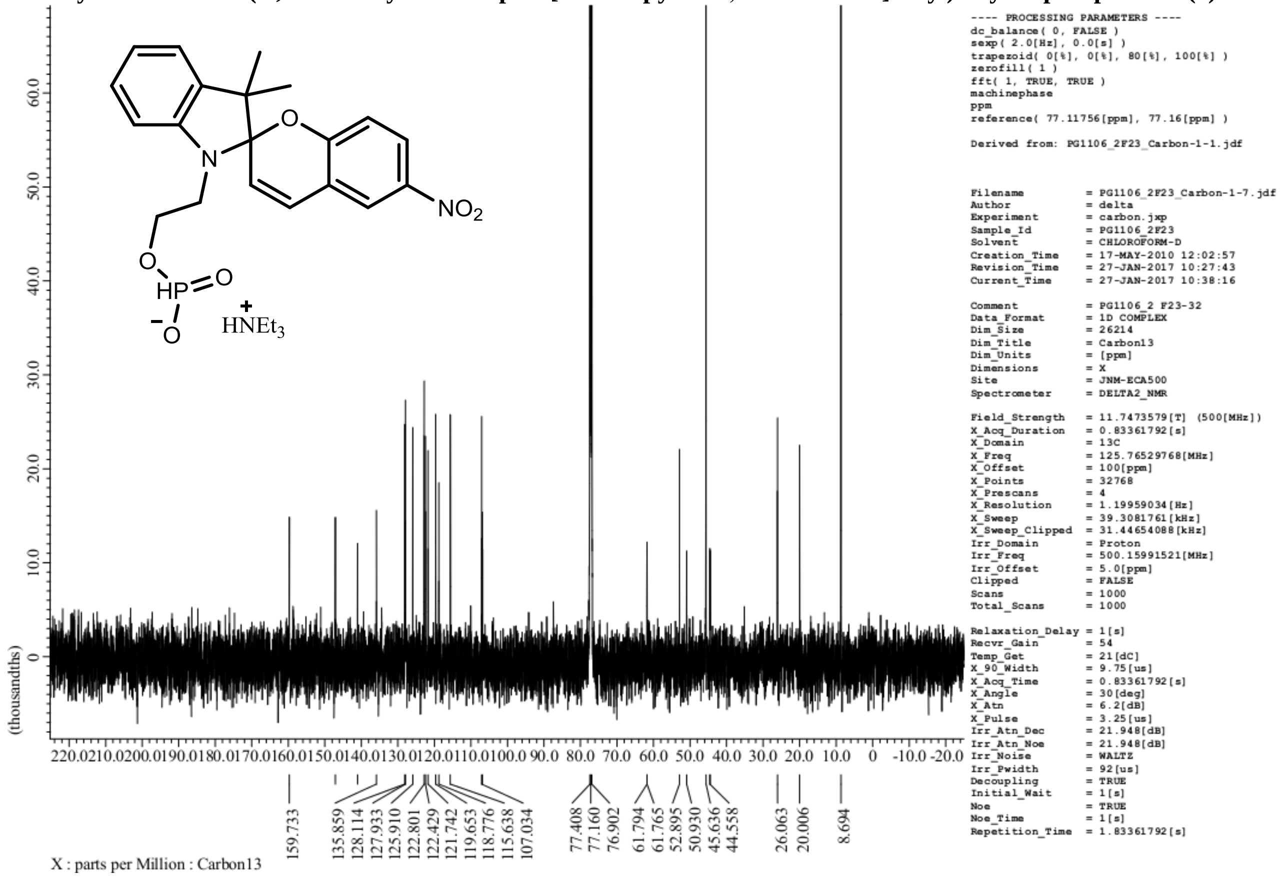

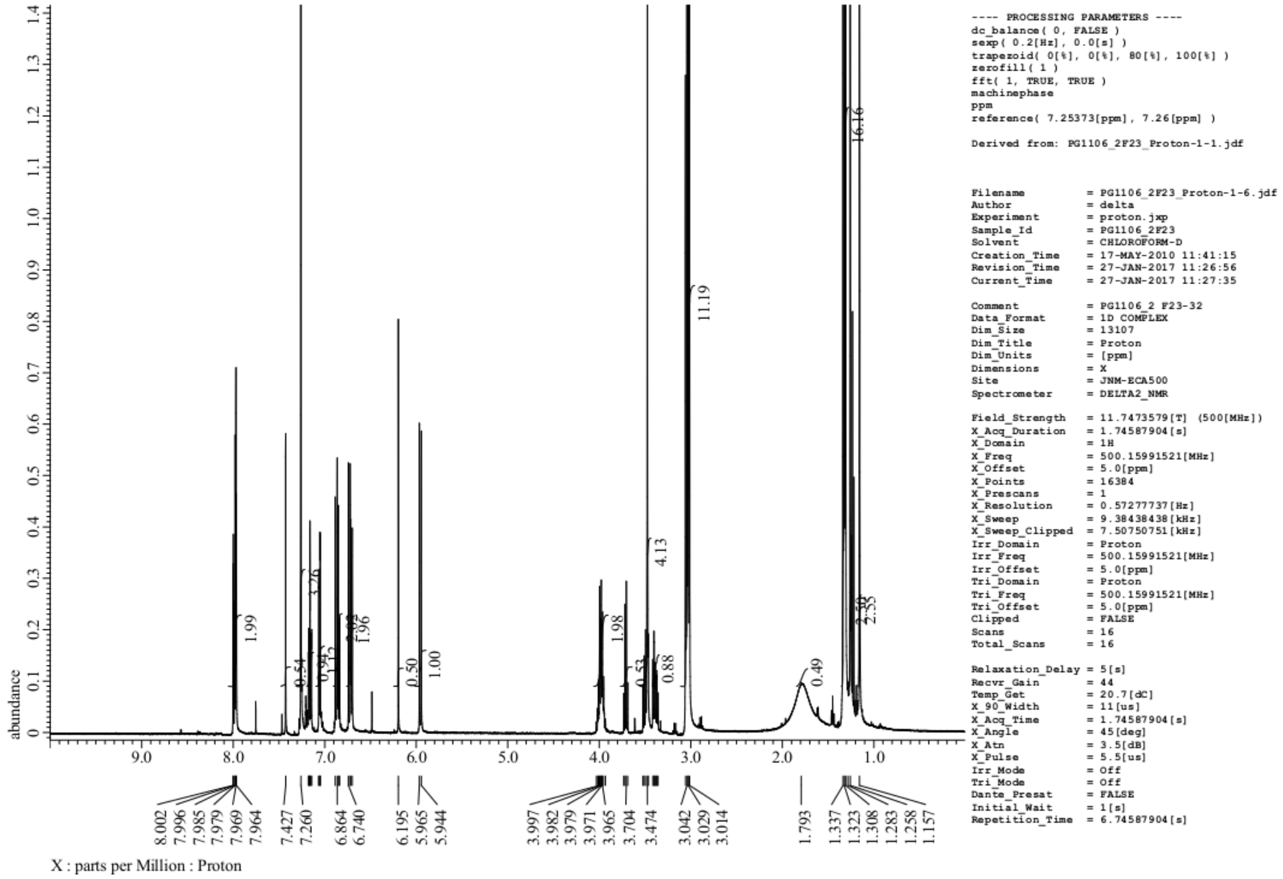

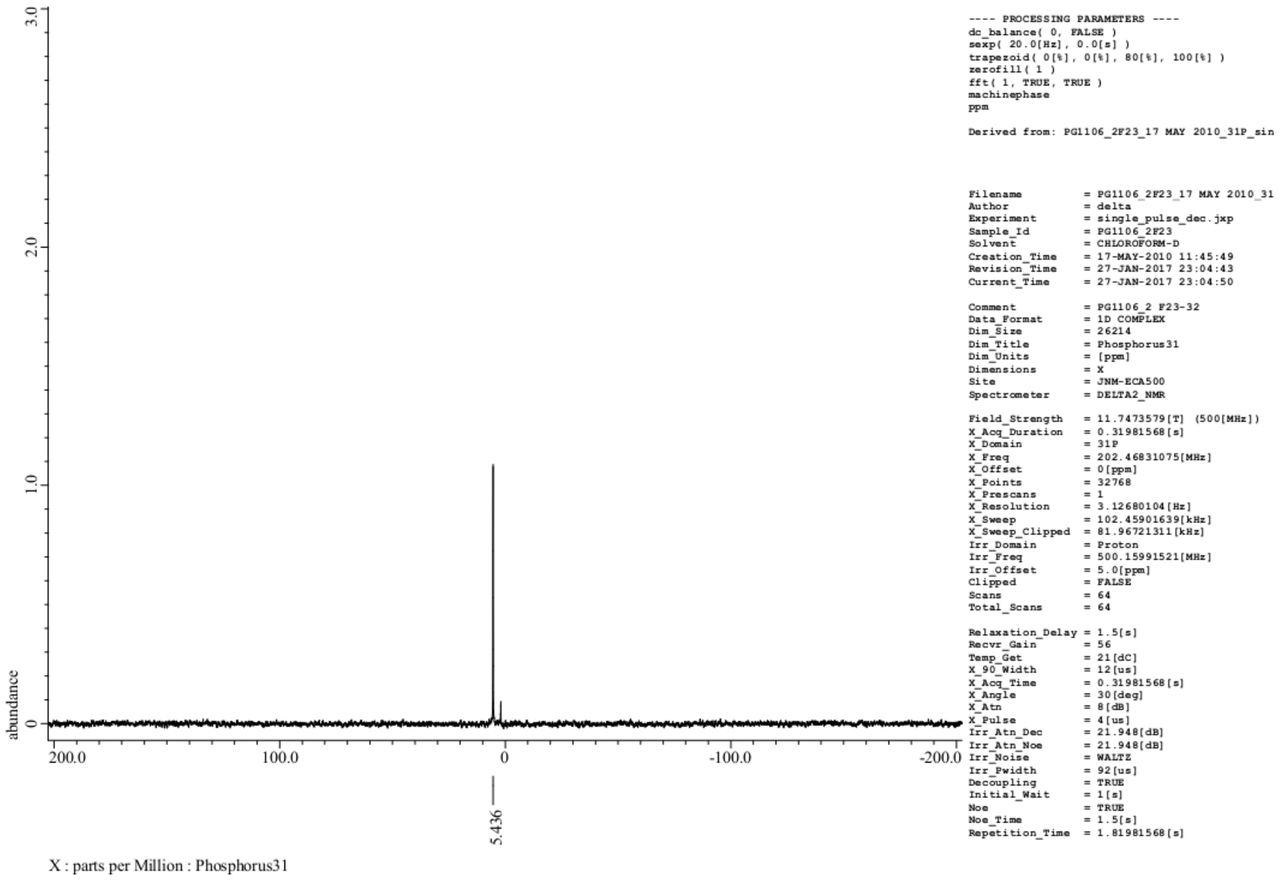

Triethylammonium ((R)-2,2-dimethyl-1,3-dioxolan-4-yl)methyl (2-(3',3'-dimethyl-6-nitrospiro[1-benzopyran-2,2'-1*H*-indolin]-1'-yl)ethyl) phosphate (3)

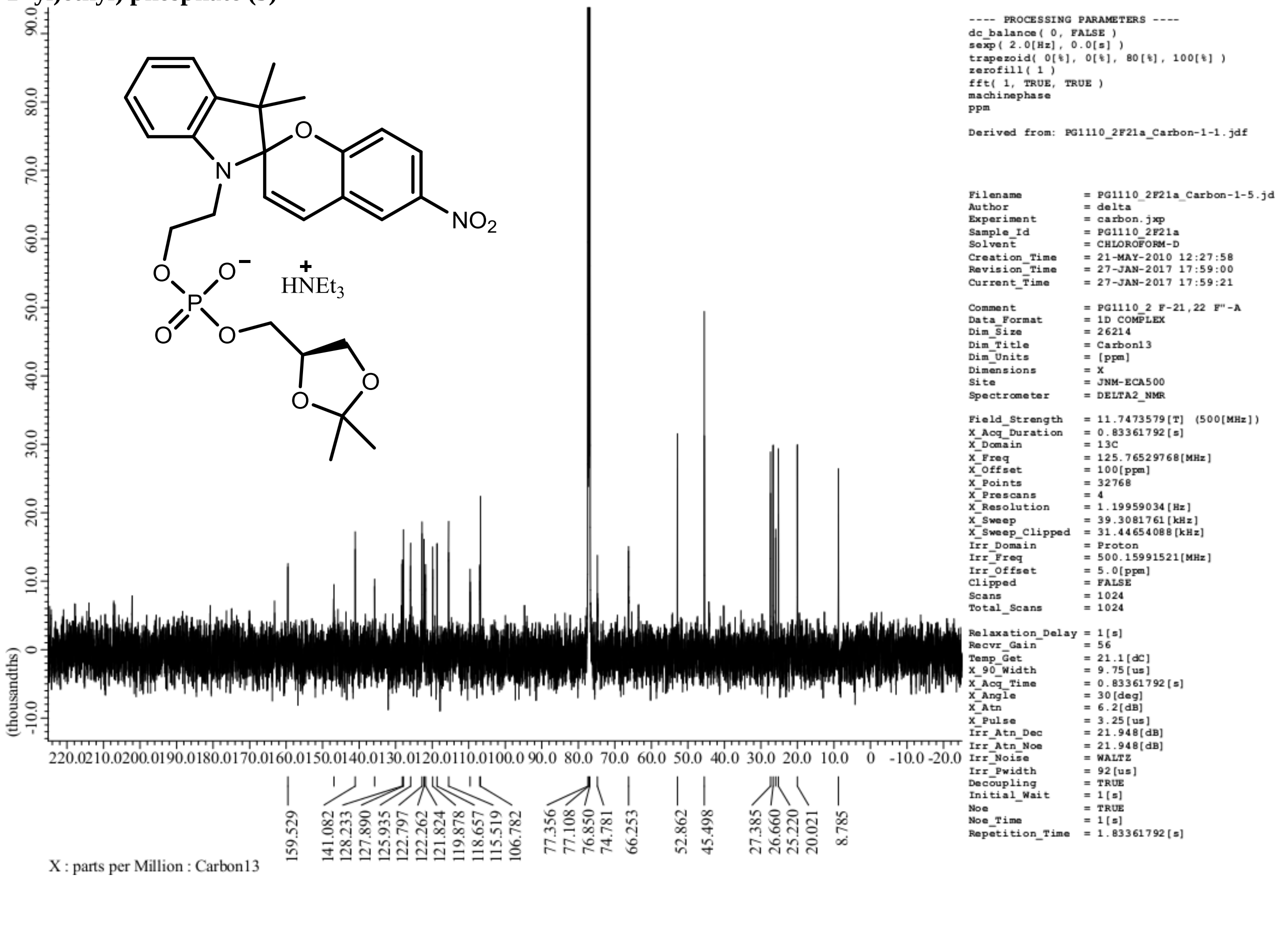

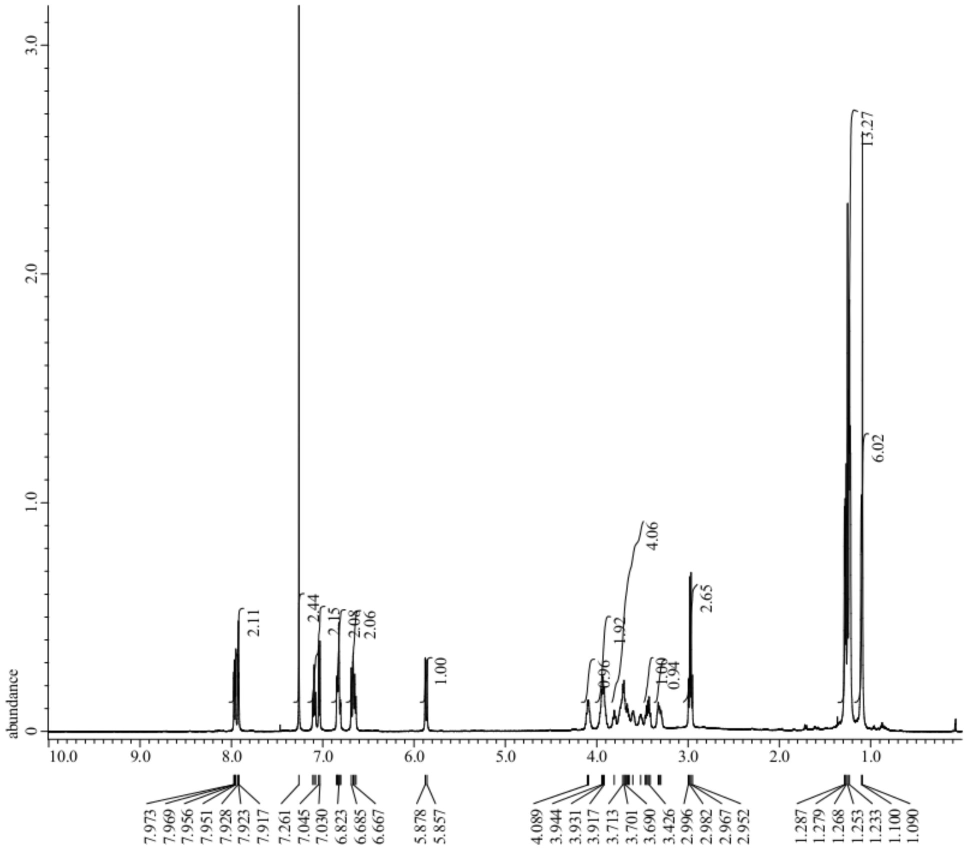

X : parts per Million : Proton

```
---- PROCESSING PARAMETERS ----
dc_balance( 0, FALSE )
sexp( 0.2[Hz], 0.0[s] )
trapezoid( 0[%], 0[%], 80[%], 100[%] )
zerofill( 1 )
fft( 1, TRUE, TRUE )
machinephase
ppm

Derived from: PG1110_2F21a_Proton-1-1.jdf

Filename         = PG1110_2F21a_Proton-1-4.jd
Author           = delta
Experiment       = proton.jxp
Sample_Id        = PG1110_2F21a
Solvent          = CHLOROFORM-D
Creation_Time    = 21-MAY-2010 12:17:38
Revision_Time    = 27-JAN-2017 23:01:49
Current_Time     = 27-JAN-2017 23:02:06

Comment          = PG1110_2 F-21,22 F"-A
Data_Format      = 1D COMPLEX
Dim_Size         = 13107
Dim_Title        = Proton
Dim_Units        = [ppm]
Dimensions       = X
Site             = JNM-ECA500
Spectrometer     = DELTA2_NMR

Field_Strength   = 11.7473579[T] (500[MHz])
X_Acq_Duration   = 1.74587904[s]
X_Domain         = 1H
X_Freq           = 500.15991521[MHz]
X_Offset         = 5.0[ppm]
X_Points         = 16384
X_Prescans       = 1
X_Resolution     = 0.57277737[Hz]
X_Sweep          = 9.38438438[kHz]
X_Sweep_Clipped  = 7.50750751[kHz]
Irr_Domain       = Proton
Irr_Freq         = 500.15991521[MHz]
Irr_Offset       = 5.0[ppm]
Tri_Domain       = Proton
Tri_Freq         = 500.15991521[MHz]
Tri_Offset       = 5.0[ppm]
Clipped          = FALSE
Scans            = 16
Total_Scans      = 16

Relaxation_Delay = 5[s]
Recvr_Gain       = 44
Temp_Get         = 20.8[dC]
X_90_Width       = 11[us]
X_Acq_Time       = 1.74587904[s]
X_Angle          = 45[deg]
X_Atn            = 3.5[dB]
X_Pulse          = 5.5[us]
Irr_Mode         = Off
Tri_Mode         = Off
Dante_Presat     = FALSE
Initial_Wait     = 1[s]
Repetition_Time  = 6.74587904[s]
```

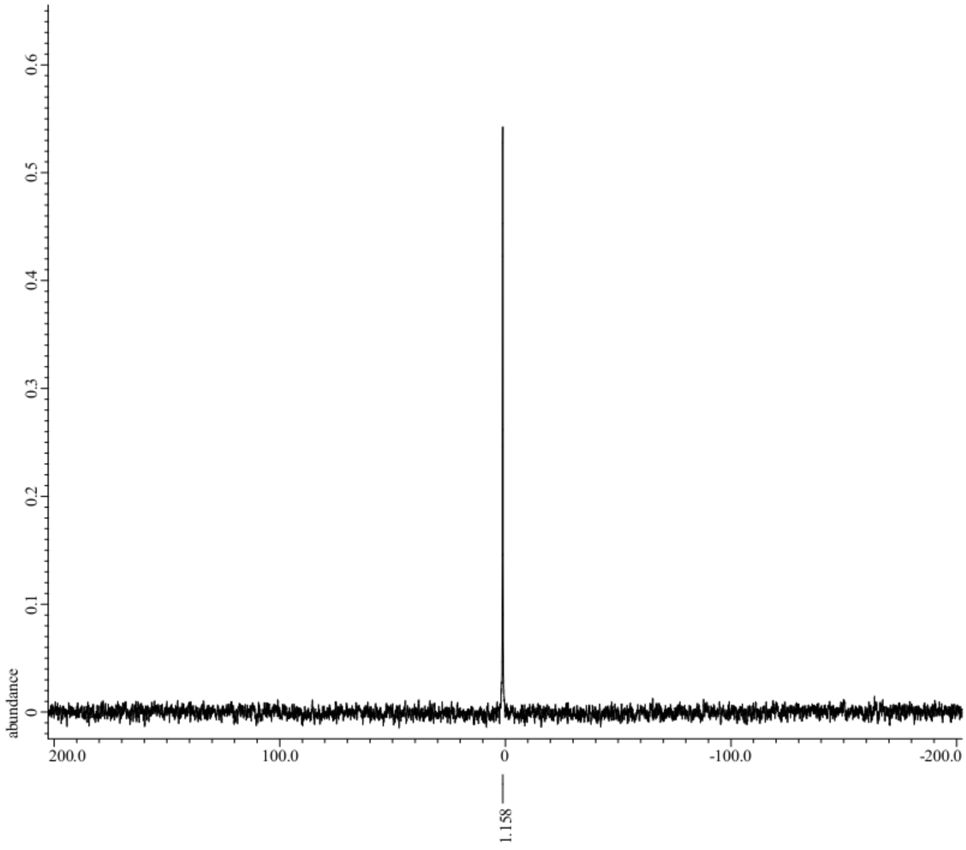

X : parts per Million : Phosphorus31

```
---- PROCESSING PARAMETERS ----
dc_balance( 0, FALSE )
sexp( 20.0[Hz], 0.0[s] )
trapezoid( 0[%], 0[%], 80[%], 100[%] )
zerofill( 1 )
fft( 1, TRUE, TRUE )
machinephase
ppm

Derived from: PG1110_2F21a_21 MAY 2010_31P_si

Filename         = PG1110_2F21a_21 MAY 2010_3
Author           = delta
Experiment       = single_pulse_dec.jxp
Sample_Id        = PG1110_2F21a
Solvent          = CHLOROFORM-D
Creation_Time    = 21-MAY-2010 12:22:12
Revision_Time    = 27-JAN-2017 16:19:43
Current_Time     = 27-JAN-2017 16:19:58

Comment          = PG1110_2 F-21,22 F"-A
Data_Format      = 1D COMPLEX
Dim_Size         = 26214
Dim_Title        = Phosphorus31
Dim_Units        = [ppm]
Dimensions       = X
Site             = JNM-ECA500
Spectrometer     = DELTA2_NMR

Field_Strength   = 11.7473579[T] (500[MHz])
X_Acq_Duration   = 0.31981568[s]
X_Domain         = 31P
X_Freq           = 202.46831075[MHz]
X_Offset         = 0[ppm]
X_Points         = 32768
X_Prescans       = 1
X_Resolution     = 3.12680104[Hz]
X_Sweep          = 102.45901639[kHz]
X_Sweep_Clipped  = 81.96721311[kHz]
Irr_Domain       = Proton
Irr_Freq         = 500.15991521[MHz]
Irr_Offset       = 5.0[ppm]
Clipped          = FALSE
Scans            = 128
Total_Scans      = 128

Relaxation_Delay = 1.5[s]
Recvr_Gain       = 56
Temp_Get         = 21.1[dC]
X_90_Width       = 12[us]
X_Acq_Time       = 0.31981568[s]
X_Angle          = 30[deg]
X_Atn            = 8[dB]
X_Pulse          = 4[us]
Irr_Atn_Dec      = 21.948[dB]
Irr_Atn_Noe      = 21.948[dB]
Irr_Noise        = WALTZ
Irr_Pwidth       = 92[us]
Decoupling       = TRUE
Initial_Wait     = 1[s]
Noe              = TRUE
Noe_Time         = 1.5[s]
Repetition_Time  = 1.81981568[s]
```

Triethylammonium ((R)-2,3-dihydroxypropyl (2-(3',3'-dimethyl-6-nitrospiro[1-benzopyran-2,2'-1*H*-indolin]-1'-yl)ethyl) phosphate (4a)

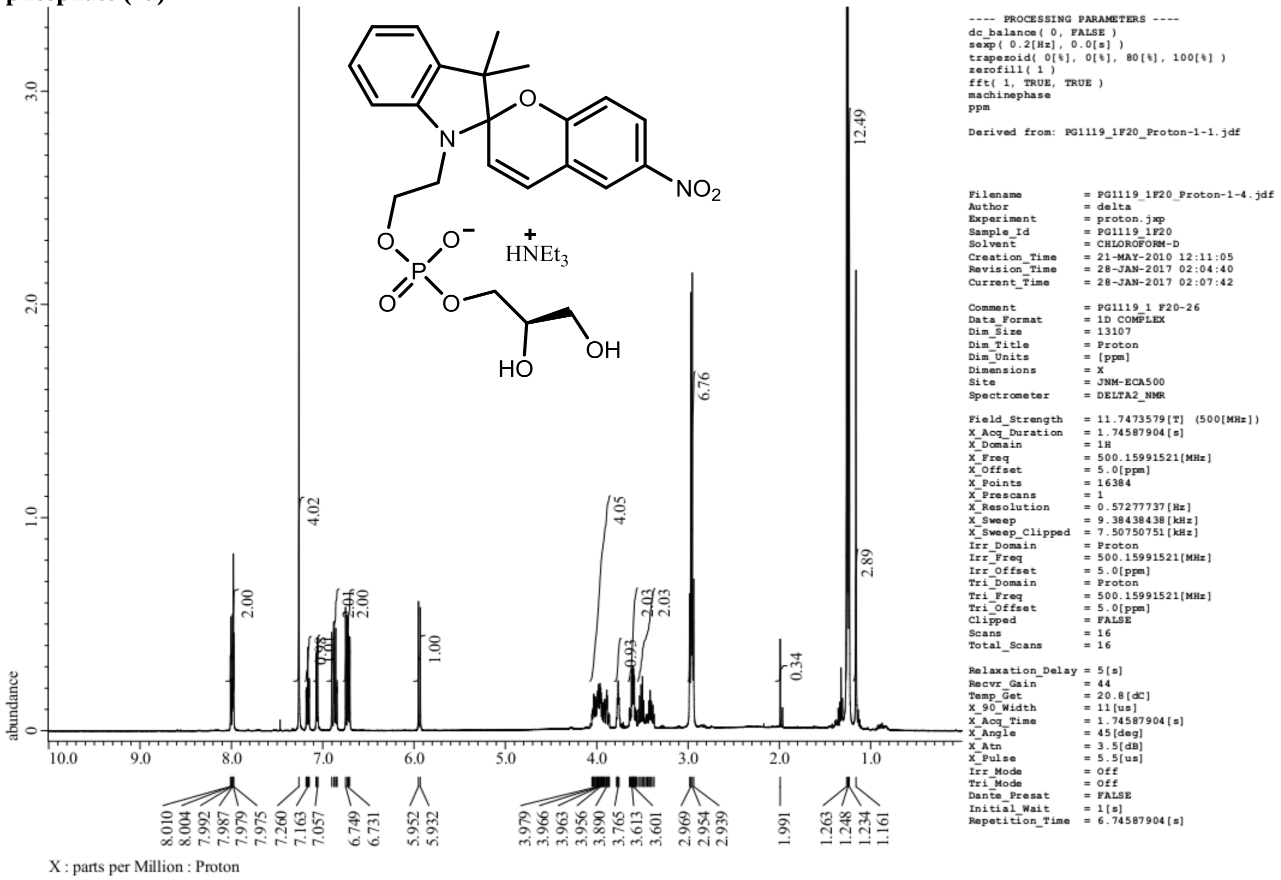

1,2-dipalmitoyl-sn-glycero-3-phosphoryl-2-(3',3'-dimethyl-6-nitrospiro[1-benzopyran-2,2'-1*H*-indolin]-1'-yl)ethane (4)

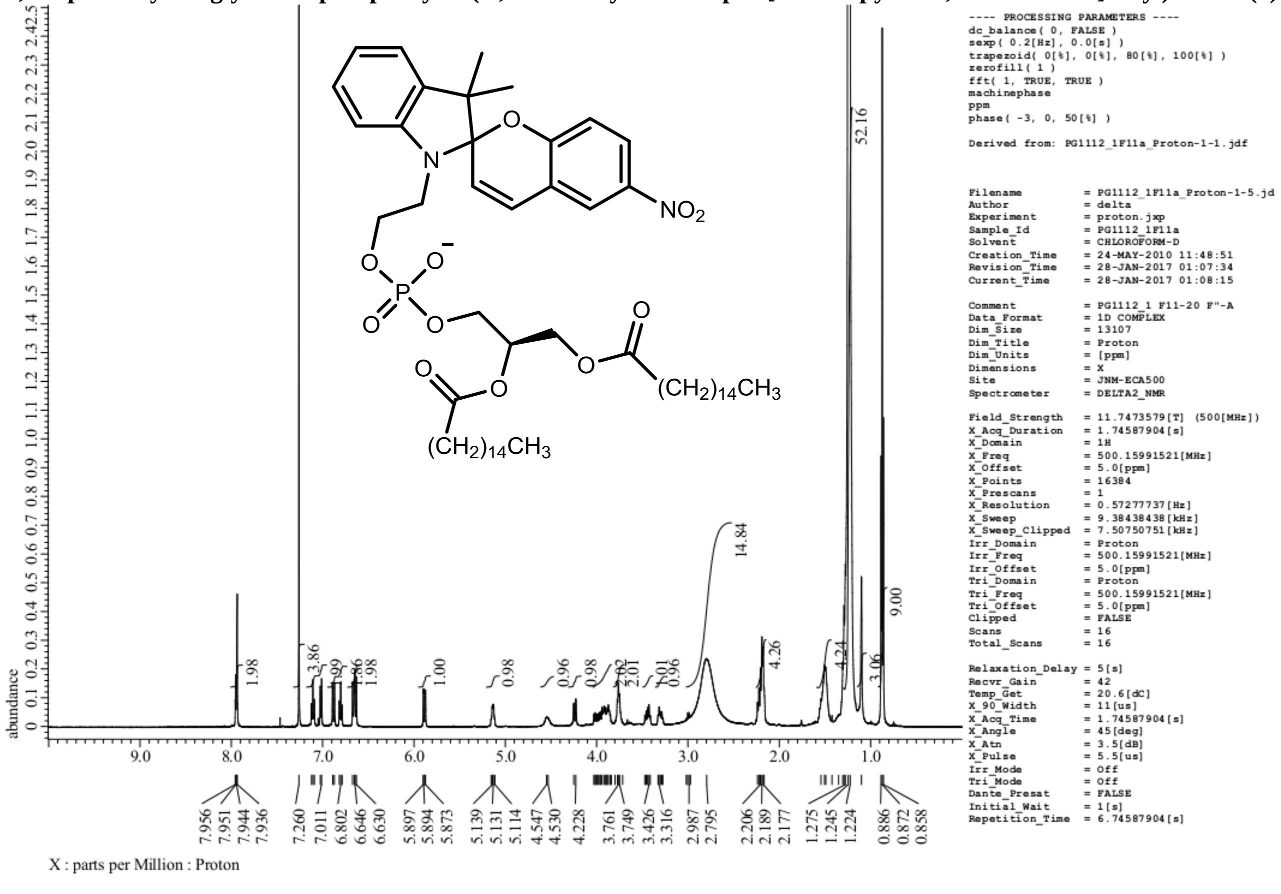

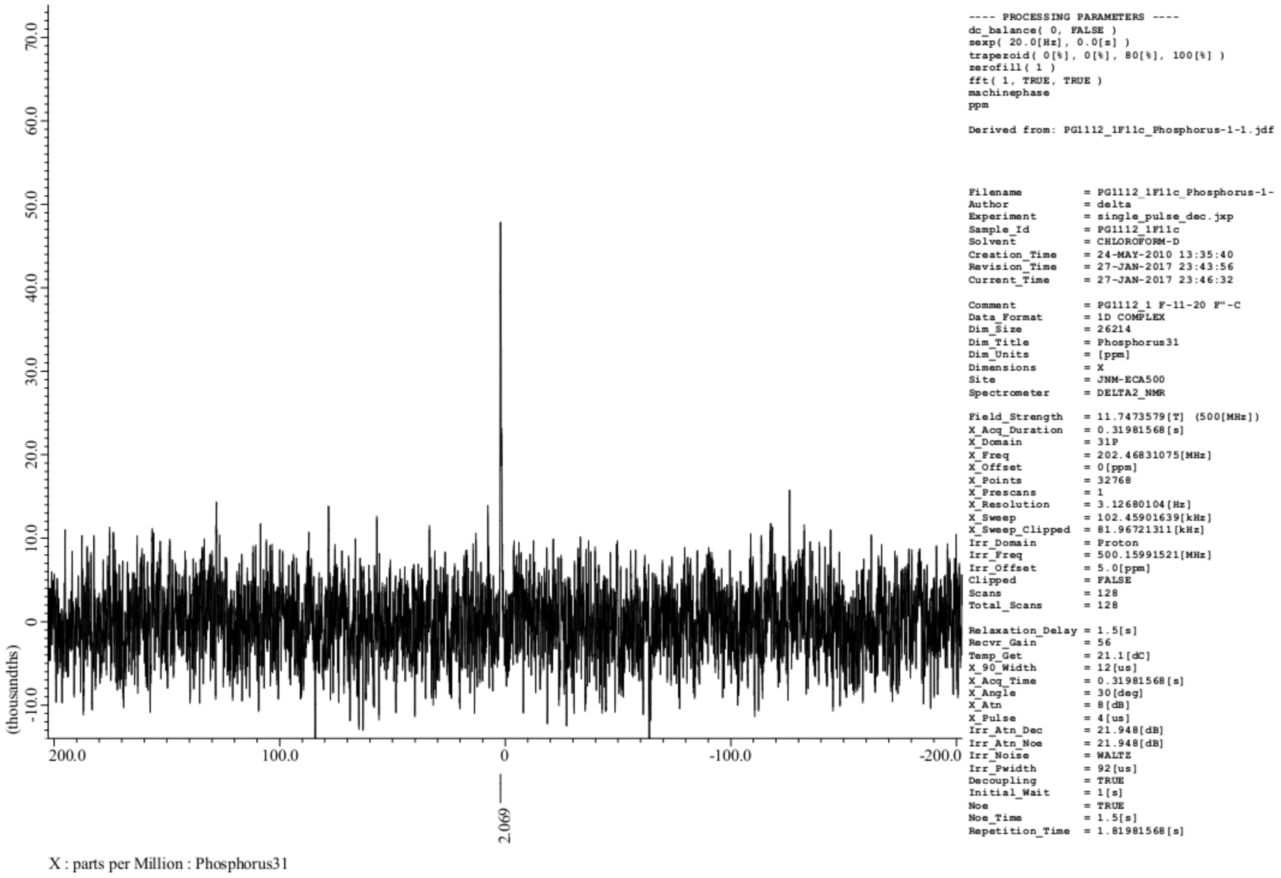
(thousandths)
X : parts per Million : Phosphorus31
2.069
---- PROCESSING PARAMETERS ----
dc_balance(0, FALSE)
sexp(20.0[Hz], 0.0[s])
trapezoid(0[%], 0[%], 80[%], 100[%])
zerofill(1)
fft(1, TRUE, TRUE)
machinephase
ppm
Derived from: PG1112_1F11c_Phosphorus-1-1.jdf
Filename = PG1112_1F11c_Phosphorus-1-
Author = delta
Experiment = single_pulse_dec.jxp
Sample_Id = PG1112_1F11c
Solvent = CHLOROFORM-D
Creation_Time = 24-MAY-2010 13:35:40
Revision_Time = 27-JAN-2017 23:43:56
Current_Time = 27-JAN-2017 23:46:32
Comment = PG1112_1 F-11-20 F"-C
Data_Format = 1D COMPLEX
Dim_Size = 26214
Dim_Title = Phosphorus31
Dim_Units = [ppm]
Dimensions = X
Site = JNM-ECA500
Spectrometer = DELTA2_NMR
Field_Strength = 11.7473579[T] (500[MHz])
X_Acq_Duration = 0.31981568[s]
X_Domain = 31P
X_Freq = 202.46831075[MHz]
X_Offset = 0[ppm]
X_Points = 32768
X_Prescans = 1
X_Resolution = 3.12680104[Hz]
X_Sweep = 102.45901639[kHz]
X_Sweep_Clipped = 81.96721311[kHz]
Irr_Domain = Proton
Irr_Freq = 500.15991521[MHz]
Irr_Offset = 5.0[ppm]
Clipped = FALSE
Scans = 128
Total_Scans = 128
Relaxation_Delay = 1.5[s]
Recvr_Gain = 56
Temp_Get = 21.1[dC]
X_90_Width = 12[us]
X_Acq_Time = 0.31981568[s]
X_Angle = 30[deg]
X_Atn = 8[dB]
X_Pulse = 4[us]
Irr_Atn_Dec = 21.948[dB]
Irr_Atn_Noe = 21.948[dB]
Irr_Noise = WALTZ
Irr_Pwidth = 92[us]
Decoupling = TRUE
Initial_Wait = 1[s]
Noe = TRUE
Noe_Time = 1.5[s]
Repetition_Time = 1.81981568[s]

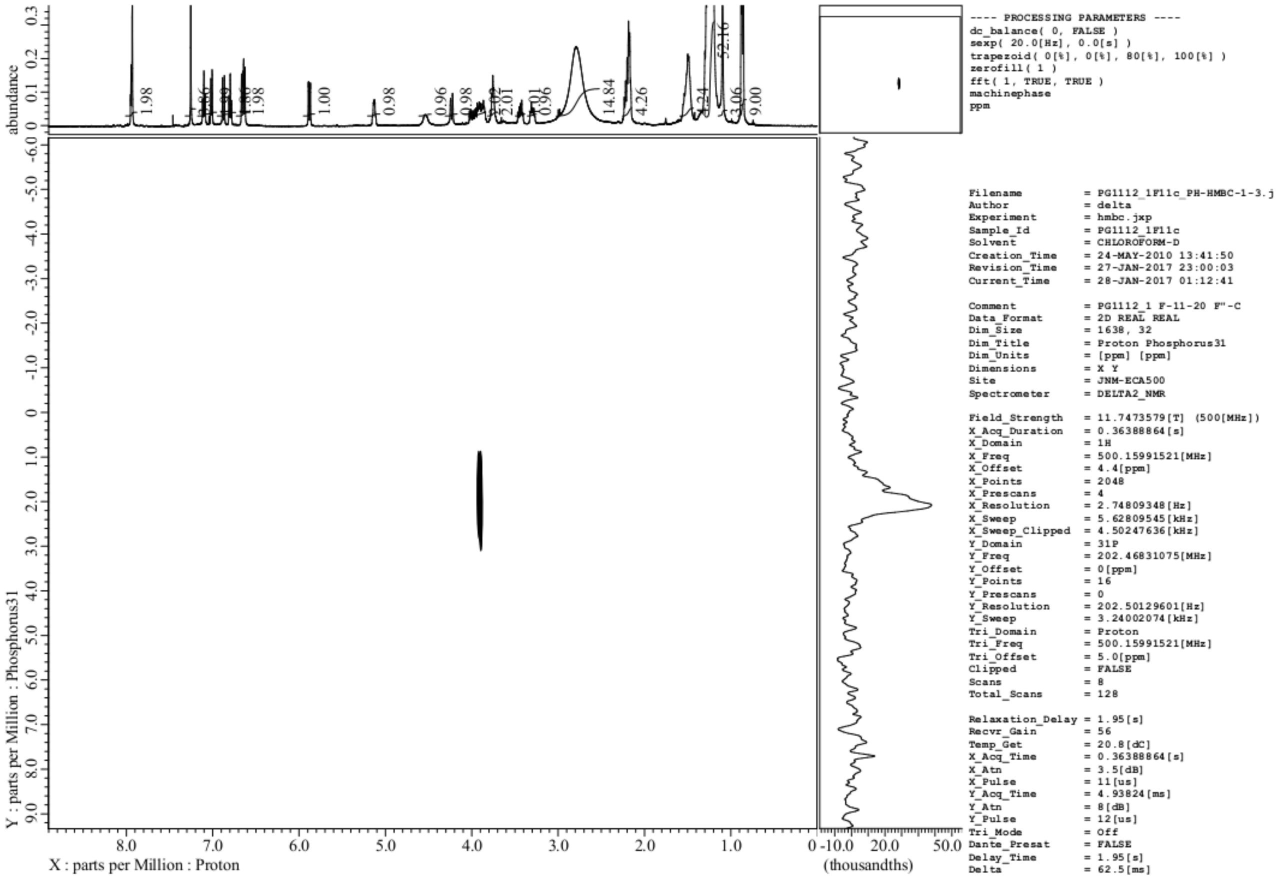
abundance
1.98
1.86
1.98
1.00
0.98
0.96
0.98
2.01
0.96
14.84
4.26
52.16
3.06
9.00
Y : parts per Million : Phosphorus31
X : parts per Million : Proton
(thousandths)
---- PROCESSING PARAMETERS ----
dc_balance(0, FALSE)
sexp(20.0[Hz], 0.0[s])
trapezoid(0[%], 0[%], 80[%], 100[%])
zerofill(1)
fft(1, TRUE, TRUE)
machinephase
ppm
Filename = PG1112_1F11c_PH-HMBC-1-3.j
Author = delta
Experiment = hmbc.jxp
Sample_Id = PG1112_1F11c
Solvent = CHLOROFORM-D
Creation_Time = 24-MAY-2010 13:41:50
Revision_Time = 27-JAN-2017 23:00:03
Current_Time = 28-JAN-2017 01:12:41
Comment = PG1112_1 F-11-20 F"-C
Data_Format = 2D REAL REAL
Dim_Size = 1638, 32
Dim_Title = Proton Phosphorus31
Dim_Units = [ppm] [ppm]
Dimensions = X Y
Site = JNM-ECA500
Spectrometer = DELTA2_NMR
Field_Strength = 11.7473579[T] (500[MHz])
X_Acq_Duration = 0.36388864[s]
X_Domain = 1H
X_Freq = 500.15991521[MHz]
X_Offset = 4.4[ppm]
X_Points = 2048
X_Prescans = 4
X_Resolution = 2.74809348[Hz]
X_Sweep = 5.62809545[kHz]
X_Sweep_Clipped = 4.50247636[kHz]
Y_Domain = 31P
Y_Freq = 202.46831075[MHz]
Y_Offset = 0[ppm]
Y_Points = 16
Y_Prescans = 0
Y_Resolution = 202.50129601[Hz]
Y_Sweep = 3.24002074[kHz]
Tri_Domain = Proton
Tri_Freq = 500.15991521[MHz]
Tri_Offset = 5.0[ppm]
Clipped = FALSE
Scans = 8
Total_Scans = 128
Relaxation_Delay = 1.95[s]
Recvr_Gain = 56
Temp_Get = 20.8[dC]
X_Acq_Time = 0.36388864[s]
X_Atn = 3.5[dB]
X_Pulse = 11[us]
Y_Acq_Time = 4.93824[ms]
Y_Atn = 8[dB]
Y_Pulse = 12[us]
Tri_Mode = Off
Dante_Presat = FALSE
Delay_Time = 1.95[s]
Delta = 62.5[ms]
